# *Tityus serrulatus* Scorpion Venom: In Vitro Tests and Their Correlation with In Vivo Lethal Dose Assay

**DOI:** 10.3390/toxins9120380

**Published:** 2017-11-23

**Authors:** Daniela Cajado-Carvalho, Juliana Galvão, Alexandre K. Kuniyoshi, Patrícia dos Santos Carneiro, Adriana Franco Paes Leme, Bianca Alves Pauletti, Eliana Blini Marengo, Fernanda V. Portaro

**Affiliations:** 1 Immunochemistry Laboratory, Butantan Institute, CEP 05503-900 São Paulo, Brazil; alexandre.kuniyoshi@butantan.gov.br; 2 Analytical Development Laboratory, Butantan Institute, CEP 05503-900 São Paulo, Brazil; juliana.galvao@butantan.gov.br (J.G.); patricia.carneiro@butantan.gov.br (P.S.C.); eliana.marengo@butantan.gov.br (E.B.M.); 3 Mass Spectrometry Laboratory, Brazilian Biosciences National Laboratory, CEP 13083-970 LNBio, Brazil; adriana.paesleme@lnbio.cnpem.br (A.F.P.L.); bianca.pauletti@lnbio.cnpem.br (B.A.P.)

**Keywords:** *Tityus serrulatus*, venom quality, toxicity, proteases, RP-HPLC, in vitro assays, median lethal dose

## Abstract

Scorpion stings are the main cause of human envenomation in Brazil and, for the treatment of victims, the World Health Organization (WHO) recommends the use of antivenoms. The first step to achieve effective antivenom is to use a good quality venom pool and to evaluate it, with LD_50_ determination as the most accepted procedure. It is, however, time-consuming and requires advanced technical training. Further, there are significant ethical concerns regarding the number of animals required for testing. Hence, we investigated the correspondence between LD_50_ results, in vitro assays, and a strong correlation with proteolytic activity levels was observed, showing, remarkably, that proteases are potential toxicity markers for *Tityus serrulatus* venom. The comparison of reversed-phase chromatographic profiles also has a potential application in venoms’ quality control, as there were fewer neurotoxins detected in the venom with high LD_50_ value. These results were confirmed by mass spectrometry analysis. Therefore, these methods could precede the LD_50_ assay to evaluate the venom excellence by discriminating—and discarding—poor-quality batches, and, consequently, with a positive impact on the number of animals used. Notably, proposed assays are fast and inexpensive, being technically and economically feasible in *Tityus serrulatus* venom quality control to produce effective antivenoms.

## 1. Introduction

A high incidence of envenomation accidents caused by animals is recorded every year in Brazil. According to Brazil’s Ministry of Health, venomous animals were responsible for more than 161,000 accidents and 244 human deaths in 2013. Specifically, cases of scorpion envenomation have been gradually increasing since 2007, representing 49% of accidents in 2013, surpassing cases of snake bites (17%) and accidents with spiders (18.5%) [1]. The main factors driving this increasing incidence are linked to the biology of scorpions, particularly associated with the parthenogenetic reproduction of the Brazilian yellow scorpion (*Tityus serrulatus*) and with its easy adaptation to urban environments [2]. These characteristics, along with the potent venom properties which contributes to the occurrence of critical clinical incidents, make the *Tityus serrulatus* scorpion one of the most significant species in terms of epidemiological and medical relevance in Brazil [3].

It is acknowledged that scorpion venom toxins mainly affect the autonomic nervous system, and, in fact, neurotoxins are the most studied molecules of scorpion venoms [4,5]. Only recently have other non-neurotoxic molecules, like peptides and enzymatic components—mainly metalloproteases and hyaluronidase—been more extensively studied [6,7,8].

The proteolytic components are considered important post-translational agents, thought to make up around 80% of the peptides present in the *T. serrulatus* venom (TsV) [9]. Other studies demonstrate that proteases from TsV can cleave human neuropeptides in vitro, and that the products of these hydrolysis may indirectly cause neurotoxic effects during the envenomation process [10,11]. Furthermore, transcriptomic studies have demonstrated that proteases are the most abundant transcripts in some scorpion venoms, such as in *T. bahiensis*, representing about 40% of the total venom [12]. On the other hand, hyaluronidases constitute around 15% of the total *T. serrulatus* venom [13], and they have already been related to venom lethality [7].

The main, and most successful, treatment for envenomation cases is through antivenom administration, a practice recommended by the World Health Organization (WHO) [14]. In Brazil, there are three producers of sera against scorpion envenoming, Butantan Institute, Vital Brazil Institute, and FUNED. Particularly, the Butantan Institute offers, through the Ministry of Health, two varieties of products free of charge to the general public. Anti-scorpion serum is produced by immunizing horses with a pool of *T. serrulatus* venom (100%). Furthermore, the Butantan Institute also produces arachnid antivenom obtained through horse immunization with a pool made up by *Tityus serrulatus* venom (53.3%) along with *Loxosceles* (26.6%) and *Phoneutria* spider venoms (20%) [15].

In order to standardize all procedures involving the production process of snake antivenoms, the World Health Organization elaborated a guideline for the quality control and management of antivenom production [14]. Although this guideline is related to the production of snake antivenoms—and because of the lack of an exclusive directive—scorpion antivenoms producers follow the same guideline. Among the crucial points to ensure high quality antivenoms by producers, quality control assays for venoms to be supplied for antivenom production are one of the key factors. The guideline recommends the determination of the median lethal dose (LD_50_) to assess venom toxicity, which is also required by the Brazilian Pharmacopoeia as a gold standard assay for venom potency determination [16]. In addition, unspecific biochemical analyses, such as SDS-PAGE gel in reducing and non-reducing conditions, determination of protein content, and size exclusion chromatography or reverse phase chromatography (RP-HPLC) are also proposed as evidence of the integrity of venom samples, and encouraged by the WHO.

Despite the in vivo method being one of the most accepted procedures to evaluate venom and antivenom potencies, objections about the use of experimental animals have been recently increasing. Some publications report that rodents do not fully replicate the human envenomation process, indicating that death assays with experimental animals as a measurement of venom efficacy, although useful, may be ambiguous [17]. Another fact of extreme importance is public opinion, which increasingly questions the scientific community regarding the possibility of replacing, reducing or, at least, refining in vivo tests through the use of alternative methods. This 3Rs approach is also encouraged by the WHO regarding LD_50_ and the median effective dose (ED_50_) protocols [14].

Notwithstanding, we should note that, to date, in vitro tests, in addition to electrophoretic analyzes and protein dosage, have not been developed for the evaluation of *T. serrulatus* venom quality. Therefore, results of in vivo tests remain the main parameter used to determine venom potency and to evaluate antivenoms efficacy.

Based on the current scenario, we evaluated the biochemical tests already proposed by the WHO and analyzed the applicability of three in vitro methods—tests of proteolytic and hyaluronidase activities, and the comparison of RP-HPLC profiles—as a quality control for *Tityus serrulatus* venom, which could precede the LD_50_ assay, discriminating venom batches with unsatisfactory quality and, consequently, reducing the number of animals required for this purpose.

## 2. Results

### 2.1. Comparison of T. serrulatus Venom Batches by Protein Content and LD_50_ Assays

The LD_50_ assay was performed for all *Tityus serrulatus* batches, as it is the gold standard test to evaluate the lethality of venom samples, and the results were used to calculate all the correlations carried out in this study.

**Figure 1:**
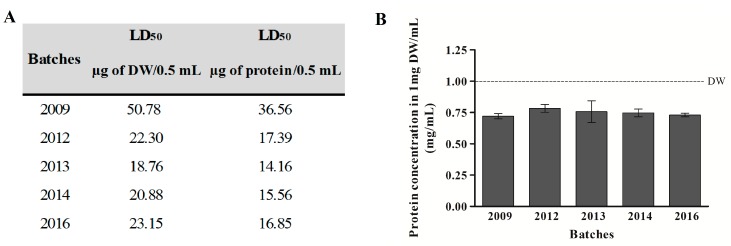
Comparative analysis of *T. serrulatus* batches for LD_50_ and protein concentration. (**A**) LD_50_ results according to DW and protein content of venom batches; and (**B**) total protein concentration into 1 mg DW/mL measured by BCA method. Three different dilutions of each batch were tested for protein quantification. Results are expressed as mean ± standard deviation, *n* = 3. DW: venom dry weight content. The dotted line indicates DW as a reference.

LD_50_ results showed that there is little variation of potency between *T. serrulatus* venom batches #2012, #2013, #2014, and #2016, exhibiting very similar values among them (mean ± SD = 15.99 ± 1.44 µg of protein/0.5 mL). However, batch #2009 showed the highest LD_50_ value (36.56 µg of protein/0.5 mL) (Figure 1A), hence, featuring poor-quality venom.

The total protein concentration of venoms was also assessed, as recommended by the WHO guideline [14]. No significant differences (mean ± SD = 0.75 ± 0.02 mg/mL) between batches included in this study were found (Figure 1B). Surprisingly, not even the TsV batch with lower potency (#2009) demonstrated altered protein content (Figure 1B). As animal venoms are not composed solely of proteins, protein concentration values obtained by the BCA methodology were proportionally lower (25%) than in dry weight venom content. However, independently of venoms’ protein content analysis—dry weight or protein quantification—both results were comparable and presented a similar ratio difference for the distinct venom batches used in the present study.

### 2.2. Electrophoretic Profile of *T. serrulatus* Venom Batches

**Figure 2:**
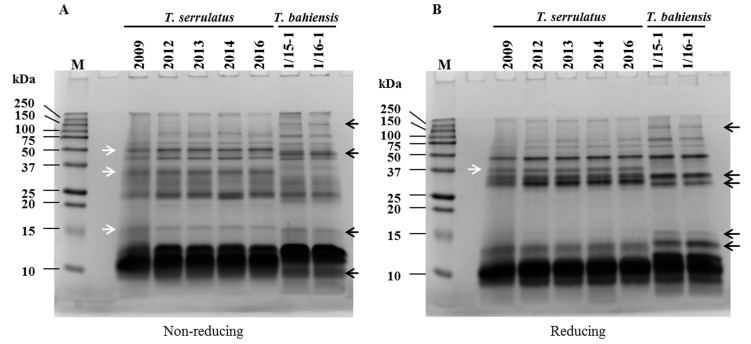
Comparison of *Tityus serrulatus* batches by 15% SDS-PAGE in non-reducing (**A**) and reducing (**B**) conditions (20 µg of protein/well, Coomassie stain). Arrows indicate visual differences between protein bands of *T. serrulatus* and *T. bahiensis* venoms. The numbers above each line indicates the batches used. M: molecular weight marker.

All TsV batches were also compared by their protein profile analysis using SDS-PAGE in reducing and non-reducing conditions (Figure 2). In addition, the capacity of this assay to distinguish protein distribution of *T. serrulatus* and *T. bahiensis* venoms was assessed. A very similar profile between all TsV batches was observed in both conditions, including batch #2009, which presented in vivo low-potency.

In order to observe exclusive interspecific characteristics, venoms of *Tityus serrulatus* and *Tityus bahiensis* were compared, and presented very similar profiles among batches, and also between the two scorpion species. Despite being genetically homologous *Tityus* species and, thus, present similar profiles in SDS-PAGE analyses, Figure 2 shows slight differences between the venoms from these two scorpions. White arrows indicate protein bands visualized only in *T. serrulatus* venom and black arrows indicate exclusive components of *T. bahiensis* venom (Figure 2). Surprisingly, this WHO recommended assay was not able to efficiently demonstrate the lower potency of batch #2009.

According to the WHO recommendation [14], a critical step for quality control is to guarantee an accurate identification of species used for venom milking. For this, SDS-PAGE was also used as a tool to distinguish *T. serrulatus* venom from others animals’ venoms. The SDS-PAGE analyses were able to discriminate *Tityus* species (*Tityus serrulatus* and *Tityus bahiensis*) from other arthropod (*Phoneutria nigriventer*, *Loxosceles gaucho*, and *Lonomia obliqua*) and snake (*Bothrops jararaca*, *Crotalus durissus terrifucus*, *Lachesis muta*, and *Micrurus frontalis*) venom samples (Supplementary Figure S1). As expected, there was a large distinction in terms of protein distribution profiles between TsV and other arthropods as well as for snake venoms (Supplementary Figure S1).

### 2.3. Reversed-Phase Chromatographic Profile Comparisosn and Peak N Content Identification by Mass Spectrometry

**Figure 3:**
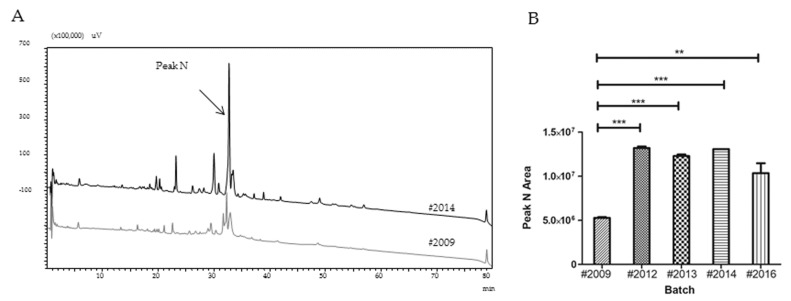
Comparative profile on reversed-phase chromatography of *T. serrulatus* (30 μg of protein) batches #2009 and #2014 (**A**), and the statistical analysis peak N area from all batches (**B**). The separations were performed at a flow rate of 0.6 mL/min using a C-8 column ACE 3 C8-300 (100 mm × 2.1 mm) and a 10–70% gradient of solvent B (being 0.1% trifluoroacetic acid (TFA) in water, as solvent A, and acetonitrile and solvent A, 9:1, as solvent B) over 70 min. In all cases, elution was followed by the measurement of ultraviolet absorption at 214 nm. One-way ANOVA was used to compare peak N areas and all batches showed statistically significant differences from batch #2009, according to the post-test Tukey’s multiple comparison (** *p* < 0.01 and *** *p* < 0.001).

Considering that primary evaluations of venom batches by their protein content and electrophoretic profile could not be related to differences observed in LD_50_ results, we continued characterizing all *T. serrulatus* venom batches (30 µg of protein per injection) by their profiles on reversed-phase chromatography (Figure 3).

Analyzing the chromatographic profiles of *T. serrulatus* venom batches, it was observed that batch #2009 had significantly lower areas for all peaks in comparison to the other batches analyzed, although all were present (Supplementary Figure S2).

Considering frequencies and average peak areas in all batches, the most prominent, named here as ‘peak N’, was chosen to carry out statistical and mass spectrometry analyses (highlighted on Figure 3A). Batch #2009 presented the most distinct value of peak N area from batches #2012, #2013, and #2014 by post-test Tukey’s multiple comparison (Figure 3B). Therefore, we selected peaks N from batches #2009 and #2014 to be analyzed by mass spectrometry, since a higher peak area could increase the probability to successfully obtain toxins identification by this procedure.

In an attempt to identify the components present in peaks N of distinct TsV batches, and to assess whether these results could be related to those obtained in the in vivo assays, the peaks N of batches #2009 and #2014 were analyzed using mass spectrometry. The results are shown in Table 1.

Peak N was characterized by the presence of hypotensins and neurotoxins (Ts1 and Ts4) on batch #2014, with a high confidence (FDR ≤ 5%). In addition, two metalloproteases (metalloserrulase 8 and Endothelin-converting enzyme 1) were also identified in #2014, indicating, once again, the importance of these enzymes for toxicity, along with two other enzymes (Cathepsin L-like and NADH-ubiquinone oxidoreductase). Batch #2009, on the other hand, was distinguished since only hypotensins were identified by mass spectrometry analysis.

### 2.4. Evaluation of Enzymatic Activities of TsV Batches

As the most common enzymes present in *T. serrulatus* venom are hyaluronidases and metalloproteases [6,10,13], these enzymatic activities of batches were tested and compared.

**Figure 4:**
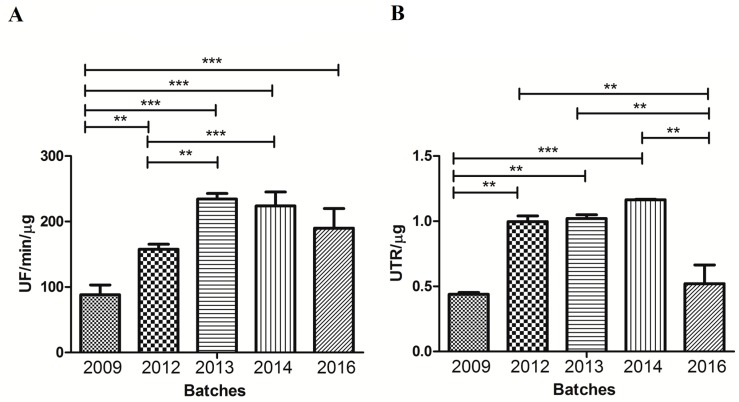
Evaluation of enzymatic activities for each *Tityus serrulatus* venom batch. (**A**) FRET-based proteolytic activity. Batches of *Tityus serrulatus* venoms (1.5 μg of protein) were incubated at 37 °C with 5 μM of the FRET substrate, in Tris 50 mM, NaCl 50 mM pH 7.4 buffer. The reactions were monitored by fluorescence (λem 420 nm and λex 320 nm) at 37 °C. The hydrolysis was expressed as units of free fluorescence per minute per µg of venom (UF/min/µg). Results are representative of three independent experiments; and (**B**) hyaluronidase activity. Batches of *Tityus serrulatus* venom (1.5 µg of protein) were incubated for 15 min at 37 °C in the presence of hyaluronic acid as substrate. After incubation, cetyltrimethylammonium bromide (2.5% *v*/*v*) was added to develop each reaction, and the resulting absorbance was measured using a spectrophotometer at λ 405 nm. Results represent the mean of three independent experiments and are expressed in units of turbidity reduction (UTR) per µg of venom, with SD. The statistical analysis was performed using 1-way ANOVA with Tukey’s Multiple Comparison Test ** *p* < 0.01 and *** *p* < 0.001).

Regarding the proteolytic activity (Figure 4, panel A), all venom batches hydrolyzed the FRET substrate. Notably, batch #2009 showed a significantly lower activity than the others, followed by batch #2012. Batches #2013, #2014, and #2016 presented statistically similar proteolytic activities values. Moreover, it was observed that all samples exhibited hyaluronidase activity (Figure 4, panel B). However, batch #2009 also had the lowest values in comparison to batches #2012, #2013, and #2014. Batch #2016 also showed a significantly lower activity when compared to batch #2014, which presents the highest hyaluronidase activity.

### 2.5. LD_50_ and In Vitro Results: Correlation Analysis

**Figure 5:**
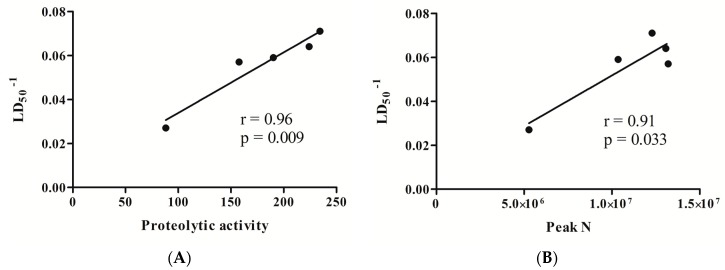
Correlation analysis of LD_50_^−1^ and proteolytic activity (**A**), and peak N area from reverse phase chromatography (RP-HPLC) (**B**) for each TsV batch. Pearson correlation analysis, presenting *p* values obtained, are shown in each panel.

Subsequently, data from all parameters analyzed in this study for each TsV batch were statistically evaluated, aiming to correlate LD_50_ values and in vitro assays. To this, LD_50_ results were normalized by the inverse of the data (LD_50_^−1^) and, subsequently, a linear dependence between LD_50_ and in vitro assays was analyzed using the Pearson Coefficient Test (Figure 5).

As shown in Figure 5A, proteolytic activity presented a remarkable correlation with LD_50_^−1^ (r = 0.96, *p* < 0.01). According to this result, batches that presented higher levels of proteolytic activity were those that presented lower values of LD_50_. Moreover, the area of RP-HPLC peak N also exhibited a very strong correlation with LD_50_^−1^ (r = 0.91; *p* < 0.05, Figure 5B). On the other hand, the correlation observed between LD_50_^−1^ and hyaluronidase activity (r = 0.73) was not statistically significant (*p* > 0.05–data not shown). Thus, both proteolytic activity levels and RP-HPLC peak N areas present a good potential of being considered markers for the definition of *T. serrulatus* venoms’ potency.

## 3. Discussion

Accidents with scorpions can cause severe manifestations in humans, and immunotherapy is widely recommended in these circumstances. In Brazil, two available antivenoms are used in case of scorpionism, the antiscorpion and antiarachnidic hyperimmune sera, and both use *Tityus serrulatus* venom on its productions. It is necessary to take into consideration the possible toxicity variation of venom batches and the importance to use good-quality venoms on antivenoms production, as they must be able to neutralize the more severe envenomation symptoms. In addition, for the evaluation of the neutralizing activity of antivenoms, a pool of good quality venom is also very important [14]. Therefore, verifying venom integrity and toxicity is momentous for the attainment of good antisera.

Producers of antivenoms are responsible for the venom quality control, which includes traceability and records of animals used for venom collection which must be representative of the intraspecific variation occurred in Brazil, as well as the biochemical and biological characterization of pooled venoms. The Butantan Institute, the main antivenom producer in Brazil, follows the guidelines established in the Brazilian Pharmacopoeia (5th edition), as well as the WHO Guidelines for the Production, Control and Regulation of Snake Antivenom Immunoglobulins [14,16]. Thereby, median lethal dose assays, associated with protein content determinations and SDS-PAGE analysis are now successfully used as methods for quality control of *Tityus serrulatus* venom. However, additional in vitro quality evaluation tests of TsV batches, which could at least reduce the number of experimental animal used would be very useful.

In addition to using the venom for antisera production , scorpion venoms have been successfully characterized using in vitro techniques by many research groups, such as chromogenic [18] and fluorogenic [6,10,11] substrates to measure enzymatic activities, electrophysiological methods [19] and different chromatographic columns that can be used to separate, identify, and quantify each component in a complex chemical mixture [7,8,11]. Despite this, until now, none of these techniques were used as possible in vitro tests to evaluate the quality of *T. serrulatus* venoms.

The inclusion of batch #2009 was critical to challenge the in vitro methods as good quality control tools, as all other batches featured guaranteed similar good qualities. Since 2012, procedures for milking, lyophilization, and storage of *T. serrulatus* venoms have been standardized in the Butantan Institute. However, batch #2009 was prepared before this period, so there are two possibilities to explain this poor quality: (a) failure in some stage in the process of this preparation; or (b) incorrect laboratory manipulation of this sample. In any case, batch #2009 resulted in venom of lower potency than regular venom samples, since it presented the higher median lethal dose in the in vivo assay. It is important to highlight that all batches used in the present work had LD_50_ values within the range described for this species so far—six to 96 µg per mouse—and studies have described some factors for *T. serrulatus* venom lethality variation. For instance, it has been demonstrated that the amount of α type toxins, and, also, geographical distribution of animals, have an important role in the variation of venom lethality tested in mice [20,21]. Notwithstanding, the animal model used can also influence the lethal dose, as it has been demonstrated that adult male rats present higher LD_50_ when compared to adult females, and that weanling rats are less resistant than adults [22]. However, differently, when mice were used as the animal model, adult females showed to be more resistant to this venom when compared to adult males [23]. In regards to human beings, based on the number of deaths recorded, the lethality of *T. serrulatus* venom is age-dependent, being higher in children under nine years of age, which stresses the importance of these accidents as a Brazilian public health problem [24].

Batch #2009 also helped us to notice that both SDS-PAGE and protein quantification were not able to differentiate the toxicity of different TsV batches. A few protein bands were visualized slightly less intensely in batch 2009 in comparison to the other batches studied. However, these results do not seem clear enough to conclude that batch #2009 shows lower in vivo toxicity. Instead, we showed that proteolytic activity measurements and RP-HPLC profile analysis presented a very strong correlation with LD_50_ values, indicating that both in vitro assays represent good indicators for TsV quality. Thus, we present, for the first time, two in vitro methodologies that can be used as minimal qualifying tests for potency evaluation of *T. serrulatus* venoms.

Metallopeptidases have attracted our attention due to their unexpected abundance in scorpion venoms, including *T. serrulatus* venom. The role of peptidases in pancreatitis caused by scorpion envenomation has also been described, with the metallopeptidase antarease being capable of degrading proteins that participate in vesicle transport [25]. Additionally, active recombinant antarease caused neuroparalysis at the neuromuscular junction of rats and flies (*D. melanogaster*) by cleaving proteins on the surface of the pre-synaptic membrane, which could be important during the envenomation process [26]. Recently, an angiotensin I-converting enzyme-like peptidase (ACE-like) was purified and characterized in the TsV, and may contribute to envenomation symptoms, especially the resulting hypertension presented by victims [11]. Additionally, in a study of proteolytic components of the *Rhopalurus junceus* scorpion [27], the low toxicity for humans of this venom correlates with its low enzymatic activity. Therefore, despite few—and recent—information on proteases as important *T. serrulatus* venom toxins, the data presented here reinforces this possibility. Our results show that while the #2009 batch simultaneously presented the lowest total proteolytic activity and worst lethal dose value, the batch with the highest toxicity, #2013, was also the one with the highest level of proteolytic activity. Thus, in addition to these results being important for future use as an analytical method, they help in better understanding the envenomation process.

The second class of enzymatic components studied in this work as a possible marker of quality control was hyaluronidases. Venom hyaluronidases were reported as important enzymatic components present in many scorpion species [27,28,29] and are also well-characterized components of *Tityus serrulatus* venom [6,7]. Originally termed as “spreading factors”, venom hyaluronidases are known to increase the permeability of toxins into tissues [4,30]. Although hyaluronidases are considered non-toxic by themselves, these molecules contribute to local and systemic envenomation [7]. In the present work, batch #2009 showed a relevant low hyaluronidase activity when compared to three other batches studied (batches #2012, #2013, and #2014), but not in comparison to batch #2016. Thus, the results concerning the hyaluronidase activity were not statistically significant, probably due to the number of venom batches used.

Undoubtedly, neurotoxins acting on sodium and potassium channels are important components of *T. serrulatus* venom [4]. Thereby, electrophysiological potential measures may also be an alternative in vitro test to verify the toxicity of venom batches rather than animal testing. In fact, a recent study indicates that antivenom efficacy evaluation by electrophysiological analysis is feasible [19]. Although we did not use this approach, two neurotoxins (Ts1 and Ts4,) were detected by MS/MS analysis as components of RP-HPLC peak N from batch #2014, but which were undetectable in the same peak form batch #2009.

Ts1 (or TsTx-I) was the first TsV characterized molecule and is considered the most abundant and best-studied neurotoxin of this venom [4]. The effects of Ts1 are associated to catecholamine releasing through Na^+^ channels with a concomitant increase in mean arterial pressure. Among other effects, Ts1 neurotoxin also increases the pancreatic exocrine secretion, and contributes to epilepsy and behavior alterations in rats [31]. The Ts4 is also a neurotoxin that affects mammalian Na^+^ channels. It has, however, low toxicity, as previous studies had classified this molecule as a non-toxic peptide [32]. On the other hand, Ts4 triggers polyclonal antibodies able to neutralize a mixture of all toxic proteins from *T. serrulatus* venom, indicating that this molecule might also be involved in the toxic action of the neurotoxins [33].

In addition to neurotoxins, two metalloendopeptidases were detected by MS/MS analysis in peak N from batch #2014, which are absent in the same peak from batch #2009, metalloserrulase 8, and endothelin-converting enzyme I. Until now, neither of the two metalloproteases was functionally studied or purified, but both were described from the TsV venom gland cDNA library [34]. Thus, the presence of these molecules on peak N from batch #2014 suggests, once more, the importance of proteases during the evaluation of venom batches quality.

In fact, the low detection of all components in batch #2009 on RP-HPLC may be due to their hydrolysis by venom enzymes, as high carboxy-, amino-, and endopeptidase activities that can degrade venom toxins had already been observed in TsV [9,10]. To our surprise, no significant differences were observed in the protein content and in the electrophoretic profile of all batches used in the present work. We believe that this fact is due to the high number of peptides present in the TsV (80%) that is hardly detectable by mentioned methodologies. Therefore, in the specific case of *T. serrulatus* venom, SDS-PAGE analysis and protein content dosages are not sufficient to be used as venom in vitro quality control. On the other hand, in vitro assays for proteolytic activity evaluation and RP-HPLC profiles comparison of *T. serrulatus* venom may be an alternative to the current potency test for the venom, especially to screen venom batch quality prior to LD_50_ testing.

In addition to providing important information about proteases as putative toxic molecules, venom evaluations using in vitro assays proposed here are faster, and technically and economically feasible. Furthermore, they show a low variability of results between assays. Another important advance is a very significant decrease—about 1000 times less—in venom consumption for these in vitro assays when compared to LD_50_ determination (Table 2). Scorpions are small animals, and extractions are laborious and of low yield, so the significant decrease in venom consumption means an equally significant decrease in the number of extractions needed.

Certainly, in vitro assays do not represent the complexity of the synergic *T. serrulatus* venom toxic effects observed in in vivo tests, as they evaluate a specific biochemical characteristic, such as proteolytic and hyaluronidase activities. Thus, it is probable that the in vitro assays will not completely end the use of animals in LD_50_ assays. However, and not only in this TsV case, the insufficient number of parallel in vitro and in vivo studies is one of the reasons why it was difficult to decrease in vivo tests. In this way, based on the satisfactory results present here, we expect that soon it will be a consistent alternative, aiming at the use of a smaller number of experimental animals.

## 4. Materials and Methods

### 4.1. Venoms

*Tityus serrulatus* venoms were identified here as #2012 (batch 01/12-1), #2013 (batch 01/13-1), #2014 (batch 01/14-1), and #2016 (a pool of batches 01/90-1 and 01/xx-1), respectively, and *Tityus bahiensis* (batches #1/15-1 and #1/16-1) venoms were provided by the Strategic Center for Venoms and Antivenoms, Butantan Institute. The *Tityus serrulatus* venom named here as #2009 was a kind gift from Dr. Ana Leonor Abrahão Nencioni, Pharmacology Laboratory, Butantan Institute. *Phoneutria nigriventer* (batch 01/15-1), *Loxosceles gaucho* (batch 2802), *Lonomia obliqua* (batch 4), *Bothrops jararaca* (batch 005), *Crotalus durissus terrifucus* (batch 02), *Lachesis muta* (batch 01/11-1), and *Micrurus frontalis* (pool composed by batches 01/11-1 and 02/11-1) venoms were provided by the Biological Quality Control Section, Butantan Institute. Lyophilized venom batches were maintained at −20 °C and were resuspended in 0.85% saline solution, pH 6.8 (in a final concentration of 2 mg of dry weight venom/mL). All venom samples were aliquoted in 20–50 µL per vial for independent testing.

### 4.2. Protein Quantification Assay

Total protein concentration for all venoms was determined using the Pierce BCA Protein Assay kit (Thermo Scientific, Waltham, MA, USA), following the manufacturer’s instructions for microplate procedures. Samples were tested at 1:2, 1:4, and 1:5 dilutions in saline solution (0.85% sodium chloride *w*/*v*, pH 6.8). The final total protein concentration of each venom batch is represented by the mean ± standard deviation of 1:2, 1:4, and 1:5 dilution results.

### 4.3. In Vivo Assay

#### 4.3.1. Animals

Male and female Swiss mice, 17–22 g, were obtained from the Central Animal Facility of the Butantan Institute and housed in the Biological Quality Control Section, Butantan Institute, for in vivo assays. The animals had ad libitum access to water and food. All experimental procedures involving animals were in accordance with ethical principles in animal research adopted by the Brazilian Society of Animal Science and the National Brazilian Legislation No. 11.794/08. Animal care and experimental procedures were approved by the Institutional Committee for the Care and Use of Laboratory Animals from Butantan Institute (CEUAIB protocol number 3060030616).

#### 4.3.2. Median Lethal Dose (LD_50_) Assay

The in vivo potency of the *Tityus serrulatus* venom (TsV) batches was tested by median lethal dose (LD_50_) assay. Tests were performed at the Biological Quality Control Section of the Butantan Institute, following Brazilian Pharmacopeia guidelines [16]. Briefly, each TsV batch was diluted to five doses, with the dilution factor being constant and no higher than 1.5 times (50.78, 33.86, 22.57, 15.05, and 10.03 µg dry weight venom/mouse), in a final volume equal to 0.5 mL (0.85% saline solution, pH 7.0) per animal. Each dose of venom was inoculated intraperitoneally into 10 mice. The number of surviving mice was counted 48 h after the venom administration. LD_50_ was performed only once for each TsV batch tested and the results were expressed as µg of dry weight venom per 0.5 mL calculated according to the statistical probit method [35] (Combistats software, version 5.0, EDQM/Council of Europe, Strasbourg, France, 2013). In parallel, LD_50_ values were also normalized for protein amount (BCA assay results) and expressed as µg of protein venom per 0.5 mL. These tests were performed according to quality control standards applying good laboratory practices.

### 4.4. SDS-PAGE Profile of Animal Venoms

Venom profile analysis was carried out by 15% SDS-PAGE technique in non-reducing and reducing (355 mM 2-mercaptoethanol, incubation at 100 °C for 10 min) conditions [36]. Twenty micrograms (BCA quantification) of each of the venoms were loaded into the gels. After electrophoresis running at 100 V for approximately 2 h, gels were stained with Coomassie G-250 Stain. Molecular weight marker (ranging 12–225 kDa; 5 µL/lane) was introduced into the first lane in all runs. All the reagents were purchased from Bio-Rad (Hercules, CA, USA): 4x Laemmli sample buffer, 10x Tris-Glycine-SDS running buffer, Bio-Safe Coomassie G-250 Stain and Precision Plus Kaleidoscope Standard.

### 4.5. Comparative Profile of *Tityus serrulatus* Venom on Reverse-Phase-HPLC

The protein profiles of all *T. serrulatus* (TsV) samples (30 µg of protein per injection) were analyzed on a reverse-phase (RP) HPLC system (Prominence, Shimadzu, Japan) at 0.1% trifluoroacetic acid (TFA) in water, as solvent A, and acetonitrile and solvent A (9:1 ratio), as solvent B. Acetonitrile and TFA were acquired from J.T. Baker. The separations were performed at a flow rate of 0.6 mL/min using a C-8 column ACE 3 C8-300 (100 mm × 2.1 mm) and a 10–70% gradient of solvent B over 70 min. In all cases, elution was followed by the measurement of ultraviolet absorption (214 nm). One-way ANOVA was used to compare the peak N areas (*p* < 0.05 for all comparison analysis between batch #2009 and the other batches, 95% confidence interval). Batches #2009 and #2014, presented high statistical difference (***), according to the post-test Tukey’s multiple comparison (*p* < 0.05, 95% confidence interval, ** *p* < 0.01 and *** *p* < 0.001). Thus, peaks Ns from #2009 and #2014 were manually collected and had their components analyzed by mass spectrometry.

### 4.6. *Tityus serrulatus* Toxins Identification

Label-free Discovery Proteomics: Peaks Ns from batches #2009 and #2014 manually collected from the HPLC system were analyzed on an ETD-enabled LTQ Orbitrap Velos Mass Spectrometer (Thermo Fisher Scientific, Waltham, MA, USA) connected to a nanoflow liquid chromatography column (LC-MS/MS) by an EASY-nLC System (Proxeon Biosystems, Odense, Denmark) through a Proxeon nanoelectrospray ion source. Peptides were separated by a 2–30% acetonitrile gradient in 0.1% formic acid using an analytical column PicoFrit Column (20 cm × ID75 µm, 5 µm particle size, New Objective, Woburn, MA, USA), at a flow of 300 nL/min over 30 min. The nanoelectrospray voltage was set to 2.2 kV, and the source temperature was 275 °C. All instrument methods for the LTQ Orbitrap Velos were set up in the data-dependent analysis (DDA) mode. The full scan MS spectra (*m*/*z* 300–1600) were acquired in the Orbitrap analyzer after accumulation to a target value of 1 × 10^6^. The resolution in the Orbitrap was set to r = 60,000. The 20 most intense peptide ions with charge states ≥2 were sequentially isolated to a target value of 5000 and fragmented in the linear ion trap by low-energy CID (normalized collision energy of 35%). The signal threshold for triggering an MS/MS event was set to 1000 counts. Dynamic exclusion was enabled with an exclusion size list of 500, an exclusion duration of 60 s, and a repeat count of 1. An activation *q* = 0.25 and an activation time of 10 ms were used.

Peak lists (msf) were generated from raw data files using Mascot Distiller v.2.4.0.0 2009 (Matrix Science Ltd., Boston, MA, USA) and searched against UNIPROT database (restricted for *Tityus serrulatus*) (110 sequences; 23183 residues) using Mascot Engine v.2.3.2 (Matrix Science Ltd.), with oxidation of methionine as the variable modification and a tolerance of 10 ppm for precursor and 0.5 Da for fragment ions. Ion scores or expected cutoff were set >21 (FDR ≤ 5%).

### 4.7. Enzymatic Activities

#### 4.7.1. Hyaluronidase Activity

Hyaluronidase activity was measured as previously described [37], with modifications. Briefly, batches of *Tityus serrulatus* venom (1.5 μg of protein) were added to a 96-well plate with 20 μL of the hyaluronic acid as a substrate (1 mg/mL) and acetate buffer (0.2 M sodium acetate-acetic acid, pH 6.0, containing 0.15 M NaCl) in a final volume of 100 μL. The mixtures were incubated for 15 min at 37 °C. After incubation, 200 μL of cetyltrimethylammonium bromide (CTAB) 2.5% in NaOH 2% was added to the samples. The absorbance was measured at λ 405 nm using a Multiskan EX spectrophotometer (Thermo Scientific, Lab Systems, Vantaa, Finland) against a blank containing 100 μL of acetate buffer and 200 μL of CTAB. All of the assays (*n* = 3) were performed in duplicate. The turbidity-reducing activity was expressed as a percentage of the remaining hyaluronic acid, relative to the absorbance of the negative control (no venom addition). Results were expressed in units of turbidity reduction (UTR) per µg of venom. The substrate and buffer salts were acquired from Sigma–Aldrich (St. Louis, MO, USA).

#### 4.7.2. Proteolytic Activity

The proteolytic activity of the *Tityus serrulatus* venom samples was determined using the fluorescence resonance energy transfer (FRET) substrate peptide Abz-GFLRRV-EDDnp. Venom samples (1.5 µg of protein) were mixed with 5 µM of FRET substrate, in Tris 50 mM, NaCl 50 mM pH 7.4 in a final volume of 100 µL. The reactions were monitored on a fluorimeter (Victor 3TM, Perkin-Elmer, MA, USA, λem 420 nm and λex 320 nm), one read per minute, with a constant temperature of 37 °C. All assays were performed in duplicate and repeated three times. Specific proteolytic activities were expressed as units of free fluorescence of the cleaved substrate per min per µg of venom (UF/min/µg). The fluorescent resonance energy transfer (FRET) substrate was kindly provided by Prof. Luiz Juliano Neto, from the Department of Biophysics of UNIFESP-EPM, São Paulo, Brazil.

### 4.8. Statistical Analysis: In Vitro and In Vivo Concordance Analysis

For correlation analysis, LD_50_ was transformed to normality by applying the inverse of the LD_50_ result (LD_50_^−1^). Subsequently, analysis of linear correlation between LD_50_^−1^ and in vitro assays was conducted using Pearson’s correlation method. The correlation strength followed Evans’ suggestion: very weak: 0.00–0.19; weak: 0.20–0.39; moderate: 0.40–0.59; strong: 0.60–0.79; and very strong: 0.80–1.00 [38]. For all statistical analyses, a coefficient interval of 95% and significance level of 0.05 was applied.

## Figures and Tables

**Table 1:** Mass spectrometry peak N results from batches #2009 and #2014. The analyses were carried out on Orbitrap without digestion. The software Mascot engine v.2.3.2 (Matrix Science Ltd., Boston, MA, USA) was used to identify molecules using the UNIPROT database restricted for *Tityus serrulatus* molecules (110 sequences). A tolerance of 10 ppm for precursor and 0.5 Da for fragment ions, and FDR ≤ 5% was applied. The peptide score was set at ≥20.

Batch	Protein	Matches	Sequences
**#2009**	P84189|Hypotensin-1	13 (7)	12 (6)
	P84190|Hypotensin-2	6 (2)	6 (2)
**#2014**	P84189|Hypotensin-1	71 (53)	31 (29)
	P84190|Hypotensin-2	14 (13)	9 (8)
	P15226|Beta-mammal/insect toxin Ts1	6 (3)	3 (3)
	A0A0K1LW71|NADH-ubiquinone oxidoreductase chain 5	1 (1)	1 (1)
	U6JM89|Cathepsin L-like cysteine peptidase	1 (1)	1 (1)
	A0A076L7Z9|Endothelin-converting enzyme 1	1 (1)	1 (1)
	O77463|Toxin Ts4	1 (1)	1 (1)
	A0A076L3I6|Metalloserrulase 8	1 (1)	1 (1)

Note: The numbers in parentheses show the result excluding duplicate matches and sequences.

**Table 2:** Cost-benefit analysis between in vivo and in vitro assays.

	In Vivo Assay	In Vitro Assays	
	Lethal Dose 50%	Proteolytic Activity	RP-HPLC *
Total execution time	~48 h	10 min	70 min
Animals required	50 mice/batch	not required	not required
Amount of venom	~1.5–2.0 mg/batch/test	1.5 µg	30 µg
Result variability	<9%	<15%	<10%

* Reverse phase chromatography.

## Supplementary Materials

The following are available online at www.mdpi.com/2072-6651/9/12/380/s1, Figure S1: Comparison of *Tityus serrulatus* venom with other arthropods and snake venoms by 15% SDS-PAGE gel in non-reducing (**A**) and reducing (**B**) conditions (20 µg/well, Coomassie stain). Figure S2: Comparative profile on reversed-phase chromatography of *T. serrulatus* batches.
