# Supplementary material for: Tityus serrulatus Scorpion Venom: In Vitro Tests and Their Correlation with In Vivo Lethal Dose Assay
Source: Toxins (Basel). 2017 Nov 23;9(12):380. doi: 10.3390/toxins9120380 (PMC5744100; doi:10.3390/toxins9120380)
Supplement: Supplementary file 1 [file toxins-09-00380-s001.pdf]

Supplementary Materials: *Tityus serrulatus* Scorpion Venom: In Vitro Tests and Its Correlation with In Vivo Lethal Dose Assay

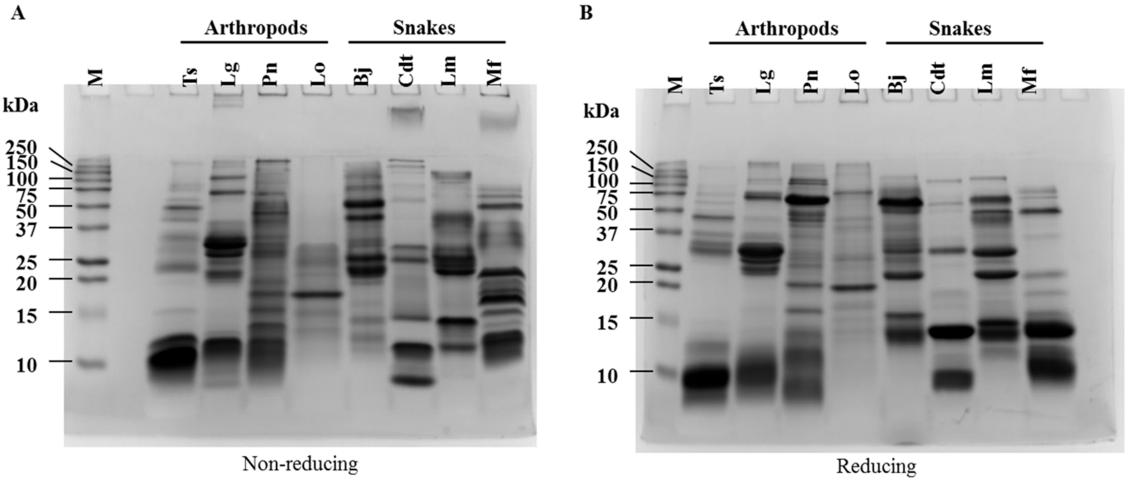

Figure S1. Comparison of Tityus serrulatus venom with other arthropods and snake venoms by 15% SDS-PAGE gel in non-reducing (**A**) and reducing (**B**) conditions (20 µg/well, coomassie stain). Ts: Tityus serrulatus, Lg: Loxosceles gaucho, Pn: Phoneutria nigriventer, Lo: Lonomia obliqua, Bj: Bothrops jararaca, Cdt: Crotalus durissus terrificus, Lm: Lachesis muta, Mf: Micrurus frontalis. M: molecular weight marker.

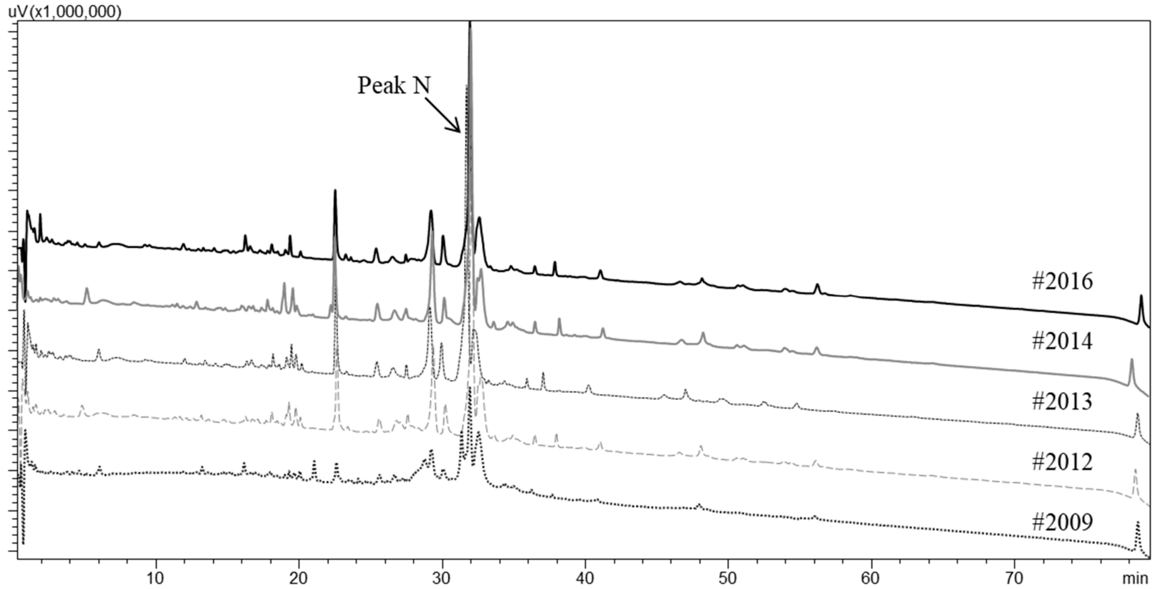

Figure S2. Comparative profile on reversed-phase chromatography of *T. serrulatus* batches (30 µg). The separations were performed at a flow rate of 0.6 mL/min using a C-8 column ACE 3 C8-300 (100 mm x 2.1 mm) and a 10–70% gradient of solvent B (being 0.1% trifluoroacetic acid (TFA) in water, as solvent A, and acetonitrile and solvent A, 9:1, as solvent B) over 70 minutes. In all cases, elution was followed by the measurement of ultraviolet absorption at 214 nm.
